# Respiratory Failure Due to a Large Mediastinal Mass in a 4-year-old Female with Blast Cell Crisis: A Case Report

**DOI:** 10.5811/cpcem.2020.5.47016

**Published:** 2020-06-19

**Authors:** Christian I. Wade, Cody J. Couperus-Mashewske, Mia E. Geurts, Nicholas Derfler, John Ngo, Kyle S. Couperus

**Affiliations:** * Uniformed Services University of the Health Sciences, School of Medicine, Department of Emergency Medicine, Bethesda, Maryland; † University of Maryland Medical Center, Department of Emergency Medicine, Baltimore, Maryland; ‡ Madigan Army Medical Center, Department of Emergency Medicine, Tacoma, Washington; § Providence St. Peter Hospital Olympia, Department of Emergency Medicine, Olympia, Washington

**Keywords:** Blast Cell Crisis, Hyperleukocytosis with Leukostasis, Thymus, Mediastinal Mass, Acute Lymphoblastic Leukemia

## Abstract

**Introduction:**

Symptomatic leukostasis is an exceptionally atypical presentation of blast crisis; and when coupled with an enlarged neoplastic mediastinal mass in a four-year-old female, an extremely rare and challenging pediatric emergency arises.

**Case Report:**

We present a unique case of a four-year-old female who arrived via emergency medical services in cardiopulmonary arrest with clinical and radiographic evidence suggestive of bilateral pneumothoraces, prompting bilateral chest tube placement. Further evaluation revealed a large mediastinal mass and a concurrent white blood cell count of 428,400 per milliliter (/mL) (4,400–12,900/mL), with a 96% blast differential, consistent with complications of T-cell acute lymphoblastic leukemia.

**Conclusion:**

This case highlights how pulmonary capillary hypoperfusion secondary to leukostasis, coupled with a ventilation/perfusion mismatch due to compression atelectasis by an enlarged thymus, resulted in this patient’s respiratory arrest. Furthermore, the case highlights how mediastinal masses in pediatric patients present potential diagnostic challenges for which ultrasound may prove beneficial.

## INTRODUCTION

Acute and chronic leukemia can both present in blast crisis resulting in hyperleukocytosis and leukostasis – an uncommon but life-threatening condition characterized by blood hyperviscosity with reduction of the other cell lines.^1^ This condition can lead up to a 40% mortality rate if not rapidly recognized and treated.^2^ Although *symptomatic* leukostasis remains extremely rare, nearly every organ system has the potential to be damaged due to the microvascular aggregates of leukocytes.^3^ Additionally, approximately 50% of patients with T-cell acute lymphoblastic leukemia were also identified to have a thymic mass.^4^–^6^ An extremely rare and challenging emergency therefore arises when pulmonary capillary hypoperfusion is coupled with compressive atelectasis by a large neoplastic mediastinal mass.

## CASE REPORT

Emergency medical services (EMS) were called to the home of a four-year-old female with a history of intermittent asthma and recent outpatient diagnosis of pneumonia after found to be cyanotic, surrounded by emesis, and without apparent respirations. Cardiopulmonary resuscitation was initiated by EMS upon arrival for pulseless electrical activity, and after eight minutes the patient achieved return of spontaneous circulation. She was subsequently intubated without the need for induction medications and transported to the emergency department (ED). Upon arrival to the ED, an initial physical exam revealed an intubated, unresponsive child with markedly diminished lung sounds bilaterally without wheezing, and an oxygen saturation of 60% on 100% fraction of inspired oxygen (FiO_2_). Additionally, respiratory therapy reported extreme difficulty with ventilation. Initial bedside chest radiographs (CXR) (Image) were obtained and showed evidence suggestive of bilateral pneumothoraces, which in conjunction with the patient’s clinical picture prompted bilateral chest tube placement. Both returned large volumes of serous fluid, although no blood or air.

Continuation of the primary survey revealed tachycardia at 132 beats per minute with strong and regular peripheral pulses in all extremities. There was no jugular venous distention. The abdomen was soft without masses. A limited neurologic exam revealed bilateral sluggish pupils, with the right pupil at 6 millimeters (mm) and the left pupil at 3 mm. There were no obvious signs of trauma, and no dermatologic findings.

After initial stabilization, collateral history was obtained from the mother who stated the patient had been feeling tired with intermittent fevers over the prior few days, which led to a diagnosis of mild viral pneumonia by her outpatient pediatrician. Per her electronic health record, this was based on the patient’s age, gradual onset of symptoms with non-toxic appearance, and non-focal pulmonary findings on auscultation. Imaging and medications were therefore deferred, but return precautions were given should the patient’s clinical presentation worsen. With regard to her asthma, the patient had only occasionally used an inhaler for night-time coughing but otherwise had never been admitted, intubated, or prescribed oral steroids. She additionally denied any other known medical conditions, surgical history, allergies, or red flags to suggest the potential for non-accidental trauma.

**CPC-EM Capsule**

What do we already know about this clinical entity?

Acute and chronic leukemia can both present in blast crisis resulting in hyperleukocytosis and leukostasis – an uncommon but life-threatening condition carrying a mortality rate as high as 40%.

What makes this presentation of disease reportable?

Symptomatic leukostasis, coupled with the presence of a large neoplastic mediastinal mass, is an exceptionally atypical, but potentially deadly, presentation of T-cell acute lymphoblastic leukemia (T-ALL).

What is the major learning point?

Pulmonary capillary hypoperfusion secondary to leukostasis, coupled with a ventilation/perfusion mismatch due to compression atelectasis by an enlarged thymus, led to this patient’s respiratory arrest.

How might this improve emergency medicine practice?

In addition to reporting an extremely rare presentation of T-ALL, this case highlights how mediastinal masses present potential diagnostic challenges for which ultrasound may prove beneficial.

Shortly thereafter, initial laboratory values resulted in a white blood cell count of 428,000 per milliliter (/mL) (4,400–12,900/mL) with a 96% blast differential, hemoglobin of 6.7 grams per deciliter (g/dL) (11.4–14.3 g/dL), and platelet count of 27,000/mL (187,000–445,000/mL). An electrolyte panel was significant for a sodium of 131 milliequivalents per liter (mEq/L) (135–145 mEq/L), potassium of 8.7 mEq/L (3.6–5.2 mEq/L), chloride of 102 mEq/L (102–112 mEq/L), bicarbonate of 10 mEq/L (19–26 mEq/L), blood urea nitrogen of 16 milligram per deciliter (mg/dL) (7–20 mg/dL), creatinine of 0.61 mg/dL (0.19–0.49 mg/dL), and a glucose of 461 mg/dL (70–140 mg/dL). An arterial blood gas resulted in a pH of 6.75 (7.35–7.45), PaCO_2_ of 60.6 mm/Hg (35–45 mmHg), PaO_2_ of 59.7 millimeters of mercury (mm Hg) (75–100 mm Hg), lactate of 12.14 mEq/L (0.2–1.8 mEq/L), HCO_3_ of 8.3 mEq/L (19–26 mEq/L), with the FiO_2_ at 100%. An alveolar-arterial gradient was calculated to be in excess of 577 mmHg (estimated normal gradient for the age of this patient is 5 mmHg).

Non-contrasted computed tomography (CT) of the head showed no evidence of intracranial abnormalities. Following the patient’s stabilization, the official read of the bedside CXR by pediatric radiology instead revealed a large mediastinal mass, manifesting features similar to that of bilateral pneumothoraces. Due to the patient’s age and concern for radiation, the patient did not receive CT of the chest as part of her initial workup. The patient was subsequently admitted for hyperleukocytosis with leukostasis and, in conjunction with hematology oncology, was started on leukapheresis and, later, induction chemotherapy. Over the course of the week, the patient’s clinical status continued to improve, and she was extubated and discharged home with a diagnosis of T-cell acute lymphoblastic leukemia.

## DISCUSSION

Given the patient’s respiratory status and what appeared to be evidence suggestive of bilateral pneumothoraces on the initial bedside CXR, bilateral chest tubes were placed. However, the official read by pediatric radiology later found the patient to have a large thymus that had features resembling what appeared to be bilateral pneumothoraces. A thymic mass is identified in 50% of patients with T-cell acute lymphoblastic leukemia, and can lead to airway obstruction.^4^–^6^ In our patient’s case, and as evidenced by an extremely elevated alveolar-arterial gradient of 577 mm Hg, her mediastinal mass likely caused compression atelectasis leading to a ventilation/perfusion mismatch, which manifested as hypoxia and respiratory distress. The mass also likely contributed to the extreme difficulty in ventilating the patient and the need for high pressures.

The thymus, proportionally the largest at birth and rather difficult to see on chest radiography by age three, lies in the anterior superior mediastinum and begins the process of involution during puberty.^7^ It normally conforms to the surrounding structures without compression or displacement; however, diffuse infiltration can cause rigidity with subsequent compression of surrounding structures.^8^ Radiologic findings consistent with a normal thymus include the following: undulating or wavy lateral margins caused by impression of overlying ribs (“wave sign”); a triangular and slightly convex right lobe due to abutting minor fissure (“sail sign”); and inspiratory and expiratory respiratory variation.^7^ Ultrasound can be particularly useful in rapidly identifying a normal vs pathologic thymus.^9^ Specifically, it should appear as a homogeneous soft tissue with finely granular echotexture and some echogenic stranding.^10^ Ultrasound can also be useful to identify ectopic thymic tissue, evaluate for focal lesions, and to differentiate from pulmonary pathology – such as verifying the presence or absence of lung sliding to rule out pneumothoraces.

Although hyperleukocytosis with leukostasis can present in a number of ways, the most common presentation is neurologic dysfunction characterized by headache, vision changes, ataxia, cranial nerve palsy, confusion, somnolence, or even coma.^3^,^11^ Emergency management of leukostasis should focus on patient stabilization, respiratory support, aggressive intravenous fluid resuscitation, prevention and treatment of disseminated intravascular coagulation, and treatment of tumor lysis syndrome should it arise. Extreme caution should be taken to avoid treatments that may increase blood viscosity, such as diuresis or red blood cell transfusions. Therapeutic leukapheresis may be indicated for patients with symptomatic hyperleukocytosis (a Grade 1B recommendation per the American Society for Apheresis), or when induction chemotherapy requires postponement. High rates of mortality are also associated with the treatment of hyperleukocytosis, and therefore immediate consultation with hematology oncology should be placed.^2^,^3^,^11^–^13^

## CONCLUSION

This case highlights the treatment of a relatively rare symptomatic hyperleukocytosis presenting with a large thymus causing mass effect and ultimately respiratory failure. We also identified potential diagnostic challenges associated with pediatric mediastinal masses, and how on review, point-of-care ultrasound may have proven more beneficial in our diagnostic approach. Specific ultrasound findings clinicians can use to identify the thymus include a homogeneous soft tissue with finely granular echotexture and echogenic stranding, and the evidence of pliability, i.e., an undulating lateral border and a triangular and slightly convex right lobe. Moreover, evidence of lung sliding can help differentiate between thymic pathology and potential pulmonary pathology such as pneumothoraces.

## Figures and Tables

**Image:**
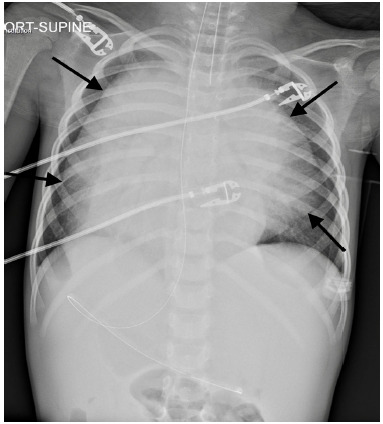
Supine portable frontal chest radiograph with arrows pointing to what resembles bilateral pneumothoraces.
